# The MAndarin spoken word—Picture IDentification test in noise—Adaptive (MAPID-A) measures subtle speech-recognition-in-noise changes and spatial release from masking in very young children

**DOI:** 10.1371/journal.pone.0209768

**Published:** 2019-01-10

**Authors:** Kevin Chi Pun Yuen, Xin Yue Qiu, Hong Yu Mou, Xin Xi

**Affiliations:** 1 Department of Special Education and Counselling, The Education University of Hong Kong, Tai Po, New Territories, Hong Kong Special Administrative Region, China; 2 Integrated Centre for Wellbeing (I-WELL), The Education University of Hong Kong, Tai Po, New Territories, Hong Kong Special Administrative Region, China; 3 Bionic Ear and Sound Technology Laboratory, Shanghai Acoustics Laboratory, Chinese Academy of Sciences, Shanghai, China; 4 Department of Otolaryngology – Head and Neck Surgery, Chinese PLA General Hospital, Beijing, China; University of Leeds, UNITED KINGDOM

## Abstract

**Background:**

Spatial release of masking (SRM) is a measure of an individual’s ability to perform speech-noise segregation, which is usually quantified by the extent of improvement of the individual’s speech recognition performance when the noise is switched from a spatially co-located position (e.g., speech and noise both presented from the front) to a spatially separated position (e.g., speech presented from the front and noise presented from the right side) with reference to the target speech. SRM is a combined measure of head shadow and binaural unmasking benefits. SRM has only been investigated in young children at group level but not at individual participant level in the international literature due to the lack of reliable speech recognition test materials able to detect subtle statistically significant within-participant changes in speech-recognition-in-noise thresholds.

**Method:**

The performance to signal-to-noise ratio (P-SNR) functions of twenty-four disyllabic words were obtained from 40 native Mandarin-speaking children aged 3.6–6.2 years with reported normal speech, language and hearing. The test items’ difficulty levels were homogenized by adjusting the speech intensity level of each item so that the adjusted signal-to-noise ratio for 50% correct score (SNR-50%) point of each item would overlap at the mean SNR-50% point of all test items. In the MAPID-A, the homogenized test items were randomly presented in an adaptive testing procedure at a fixed noise intensity level, but the speech intensity level of the upcoming test item varied in 2-dB SNR steps depending on the recognition result of the previous test item. The SNR reversal point is marked by a change from a decrease to an increase in the SNR or vice versa. Two successive SNR reversal points marked the boundaries of an excursion. The mid-points from 12 excursions (in dB SNR) were averaged to produce the adaptive SNR-50% measure (aSNR-50%).

**Results:**

The aSNR-50% results were obtained from another 12 children aged 4.8–5.3 years with reported normal speech, language and hearing. The average 99% confidence interval (CI) of all participants’ mean aSNR-50% values was ±1.61 dB SNR; therefore, 3.22 dB SNR was the average critical difference required to confirm a significant difference in the scores obtained from the same participant between two test conditions. Statistically significant within-participant SRM was identified in 95% of the participants; in other words, aSNR-50% obtained from the spatially separated condition outperformed aSNR-50% obtained from the spatially co-located condition. The adaptive testing procedure was highly reliable, with an within-participant test-retest reliability of 90.6%. and significantly limited testing time to an average of 4.2 min. This research study has fulfilled its aim on detecting subtle within-participant SRM in very young children starting from 4 years of age with a reliable statistical procedure. MAPID-A offers a reliable and efficient clinical tool to investigate speech-recognition-in-noise and SRM performances in young Mandarin-speaking children.

**Conclusions:**

The narrow CIs, high test-retest reliability, and short testing time has proven that the MAPID-A is a promising sensitive, reliable and time-efficient clinical tool to detect subtle within-participant speech-recognition-in-noise changes in children as young as 4–5 years. The MAPID-A offers a clinical tool to behaviorally track young children’s development in speech-recognition-in-noise and SRM, and to potentially review the development of the auditory neural pathway and the cerebral dominance for speech-recognition-in-noise in young children.

## Introduction

Children are much more frequently exposed to environments with interfering noises than to quiet environments [1, 2]. Accordingly, they spend long periods of time in acoustic environments where speech signals are masked by interfering sounds from multiple sources [3]. High levels of noise (i.e., unwanted sounds inside or outside a room) and reverberation (persistent sound after cessation of the source) can negatively affect speech perception, reading and spelling abilities, classroom attention, concentration and educational achievement [4]. The American-Speech-Language-Hearing Association (ASHA) (2005a) recommends that a) unoccupied classroom noise should not exceed 35 A-weighted decibels (dBA); b) the signal-to-noise ratio (SNR) should be at least +15 dB and c) unoccupied classroom reverberation times must not surpass 0.6 and 0.7 sec in smaller and larger classrooms, respectively.

In children, speech recognition performance decreases significantly when transitioning from a quiet to noisy listening environment. Compared to adults, children require a much better SNR to reach the same speech in noise detection performance (e.g. [5]) and recognize speech in noise (e.g. [6]). Many studies have reported that children’s abilities to recognize speech in noise advance with age [6–15]. The developmental trajectory of speech in noise performance was not found to plateau during early childhood, but rather to extend throughout adolescence until the individual reaches an adult level of performance [7, 11, 16].

To recognize speech in noise, children must segregate the interfering noise from the target speech to increase the perceptual salience of the target speech. The target speech-noise segregation depends strongly on the capacity of the auditory nervous system to compare subtle differences in the properties of the target speech and noise received by the ears, including the frequency spectrum, fundamental frequency, arrival time and intensity [17]. This speech-noise source segregation capacity for speech-recognition-in-noise measured by SRM is a way to reflect of the degree of maturation of the peripheral and central auditory pathways [18]. Therefore, the long developmental trajectory of speech-recognition-in-noise suggests that the peripheral and central auditory pathways do not fully mature until an individual has at least reached adolescence.

As noted above, children are continually exposed to interfering noises, and their abilities to segregate target speech from these interfering noise and to selectively attend to important information from the target speech that are critical to speech and language learning. Interfering noises include speech sounds produced by other people nearby who are not actively engaged in communication with the listener and other environmental sounds. These noises may come from the same location as the target speech (i.e., spatial co-location of the target speech and noise), such as in a classroom where a child is listening to a teacher’s instruction while the teacher simultaneously claps her hands close to her mouth. Alternatively, these interfering noises may come from locations other than that of the target speech (i.e., spatially separated target speech and noise), such as when a child is listening to the teacher’s instruction while several peers are talking simultaneously around the child. Children, not just adults, exhibit better speech recognition performance when the target speech and noise are spatially separated than when they are spatially co-located [19].

The ability of an individual to perform target speech-noise segregation can be evaluated by the extent of improvement of the individual’s speech recognition performance when the noise is switched from a spatially co-located position with reference to the target-speech (e.g., target speech and noise both from the front) to a spatially separated position (e.g., target speech from the front and noise from the side). This improvement also known as a spatial release from masking (SRM), is a collective measure of an individual’s speech-recognition-in-noise improvements from head shadow and binaural unmasking [6, 20, 21]. Regarding the head shadow, the SNRs received by the two ears differ when the target speech and noise are spatially separated (e.g., from the front and right side, respectively). By attenuating the noise coming from the right side, the head creates an acoustic shadow for the left ear, which therefore receives a better SNR, whereas the right ear is directly exposed to the noise and thus receives a poorer SNR [21, 22]. The benefit of the head shadow depends on the ability of the individual to attend more and less selectively to the information received in the ear with a higher SNR and the ear with a lower SNR, respectively. Regarding binaural unmasking, the individual’s auditory neural system computes interaural differences in the timing, phase and intensity of the target speech and noise received by the two ears. The benefit of binaural unmasking depends on the robustness of the auditory neural system for computing the interaural differences in the decorrelation of the target speech from the noise [23] to improve speech detection and recognition.

Speech recognition testing materials and procedures dedicated to the evaluation of young children’s speech-recognition-in-noise performance in spatially separated and spatially co-located noise conditions, as well as the degree of SRM, are rare, and those which are able to statistically evaluate within-participant statistical differences are even more scarce. According to the review of Yuen and Yuan [6] of previous developmental studies of SRM, a few studies reported the performance levels of young children in the aforementioned outcome measures of speech-recognition-in-noise performance; however, those procedures were conducted mainly for research purposes and limited to group performance comparisons. Nearly all previous developmental research studies of SRM offered no procedures to delineate within-subject statistical differences, thus limiting the applications of those procedures in clinical settings.

Speech-recognition-in-noise relies on the low-level auditory processing of the speech signal, as well as of high-level language and cognitive processing. Therefore, it is unsurprising that populations with identified auditory or language-based disorders would exhibit speech perception problems in noise, even if their pure tone hearing thresholds are within the normal range. For example, some children with specific language impairments [24], dyslexia [25] and auditory processing disorder (APD) [26] exhibited reduced speech-recognition-in-noise performance when compared to control groups with the same hearing sensitivity. The design of appropriate intervention programs relies critically on the delineation of the nature of the listening problems specific to each population.

The introduction of a masking noise in a listening paradigm alters the recognition performance during the listening task. The spectra and amplitude or energy relation between the speech and masking signals are important determinants of recognition performance [27]. An understanding of masked speech recognition development is important because children must learn about speech and language in natural environments that often contain multiple sources of competing sounds. As noted above, the speech-recognition-in-noise performance significantly influences the development of listening, speech and language. Therefore, the development of sensitive speech-recognition-in-noise assessment tools that could track subtle maturational changes in young children via within-participant comparisons over time is necessary. If such tools could detect subtle changes in performance within an individual participant, they could also assess the benefits of various rehabilitation procedures, such as active intervention programs for children with auditory processing disorders or the fitting of hearing aids, cochlear implants or other hearing prosthesis and assistive devices. The increased availability of resources and tools to support larger-scale developmental studies of young children could allow the determination of normative developmental benchmarks at different age ranges. These tools could then be used to identify children whose performance levels significantly fall below those of their peers, thus allowing the initiation of early intervention procedures even at young ages.

This study aims to develop a clinical tool that could be used to investigate the speech-recognition-in-noise and SRM performance levels in young children for the purpose of within-participant comparison.

## Experiment 1—Determination of the performance-SNR functions of test items and homogenization of their difficulty level

### Participants

Forty children (19 boys, 21 girls) were recruited in Beijing as study participants. All were native speakers of Mandarin. Eight children were recruited for each of five age groups corresponding to 3.5–3.9, 4.0–4.4, 4.5–4.9, 5.0–5.4 and 5.5–5.9 years of age (one child in the latter group was 6.2 years old). All groups included four boys and four girls except those corresponding to 5.0–5.4 and 5.5–5.9 years, which included five girls and three boys, and three girls and five boys, respectively. The mean age was 4.76 years (standard deviation (SD) = 0.77 years). None of the recruited children had any reported concerns with speech, language, hearing or cognitive development. Written consent was sought from the parents or guardians for the involvement of each participant in the study. This study was approved by the Human Research Ethics Committee of The Education University of Hong Kong. All participants underwent pure tone air-condition play or standard audiometry at octave frequencies of 250, 500, 1000, 2000, 4000 and 8000 Hz. All participants were found to have normal hearing sensitivities, with pure tone air condition thresholds of 15 dB HL or better in both ears at all test frequencies.

### Materials

Twenty-four disyllabic words from the *MAndarin Pediatric lexical tone and disyllabic-word Picture IDentification test in Noise (MAPPID-N)* [6, 28] were adopted. Each word was assigned to one of three test sets: (1) *“animals”*; (2) *“body parts and clothing items”* and (3) *“everyday items”*. The IPA transcriptions, written characters and English translations of these words are shown in Table 1. For this study, the materials were re-recorded by a female speaker employed as a news anchor of a TV station in Beijing, rather than using the recorded materials from the *MAPPID-N*. The recordings were collected in a sound-treated booth using a Behringer condenser microphone ECM8000, E-MU 0404 USB 2.0 Audio/ MIDI Interface, Cool Edit pro 2.0 software and a personal computer (PC). Recordings were sampled at a rate of 44,100 Hz with 16-bit resolution. During the recording, the speaker spoke the carrier phrase /ʈʂeɪˋ kɤˋtsɯˋʂɚˋ/, which means “this word is”, before the disyllabic word to standardize the overall pitch level of the speaker’s voice. A deliberate pause was introduced between the carrier phrase and the disyllabic word to facilitate the extraction of the target word without carrying over any co-articulation. Three tokens of the same disyllabic word were recorded, and only one token for each disyllabic word was selected for further processing. The naturalness and quality of the selected token were approved by two audiologists who were native Mandarin speakers. The selected tokens were subjected to low-pass filtering at 10 kHz, and the intensity levels were root-mean-square (RMS) equalized and saved individually as .*wav* files. To provide effective masking for testing in noise, a tailor-made speech spectrum of weighted noise was created according to the average speech spectrum of all tokens selected for the 24 disyllabic words. The intensity level of this noise was RMS equalized to the same level of the selected tokens and used to calibrate the presentation level.

**Table 1:** IPA transcriptions, Chinese characters and English translations of the 24 disyllabic word test items from the three test sets, which address *“animals”*, *“body parts and clothing items”* and *“everyday objects”*.

*Animals*			*Body parts and clothing items*			*Everyday objects*		
IPA	Chinese Characters	English Translation	IPA	Chinese Characters	English Translation	IPA	Chinese Characters	English Translation
/paɪˊ tʰuˋ/	白兔	Rabbit	/aɻˇ tuɔ˙/	耳朵	Ear	/tjɛnˋ ʂɚˋ/	電視	Television
/taˋ ɕjɑŋˋ/	大象	Elephant	/meɪˊmɑʊˊ/	眉毛	Eyebrow	/mɑʊˊ tɕɪnˉ/	毛巾	Towel
/kʊŋˉ tɕiˉ/	公雞	Rooster	/ʂoʊˇ tʰɑʊˋ/	手套	Glove	/ʂuˉ pɑʊˉ/	書包	School Bag
/xuˊ tjɛˊ/	蝴蝶	Butterfly	/iˉ fuˊ/	衣服	Clothing	/ʂweɪˇ peɪˉ/	水杯	Cup
/lɑʊˇ xuˇ/	老虎	Tiger	/tʰoʊˊ faˇ/	頭髮	Hair	/jɛnˇ tɕɪŋˋ/	眼鏡	Eyeglasses
/tɕʰɪŋˉ waˉ/	青蛙	Frog	/jaˊ ʈʂʰɚˇ/	牙齒	Teeth	/jɑʊˋ ʂɚ˙/	鑰匙	Key
/ɕjɑʊˇ koʊˇ/	小狗	Dog	/jɛnˇ tɕɪŋˉ/	眼睛	Eye	/jaˊ ʂwaˉ/	牙刷	Toothbrush
/ɕjʊŋˊ mɑʊˉ/	熊貓	Panda	/ʂoʊˇ pjɑʊˇ/	手錶	Watch	/yˇ sanˇ/	雨傘	Umbrella

### Methods

**Fig 1:**
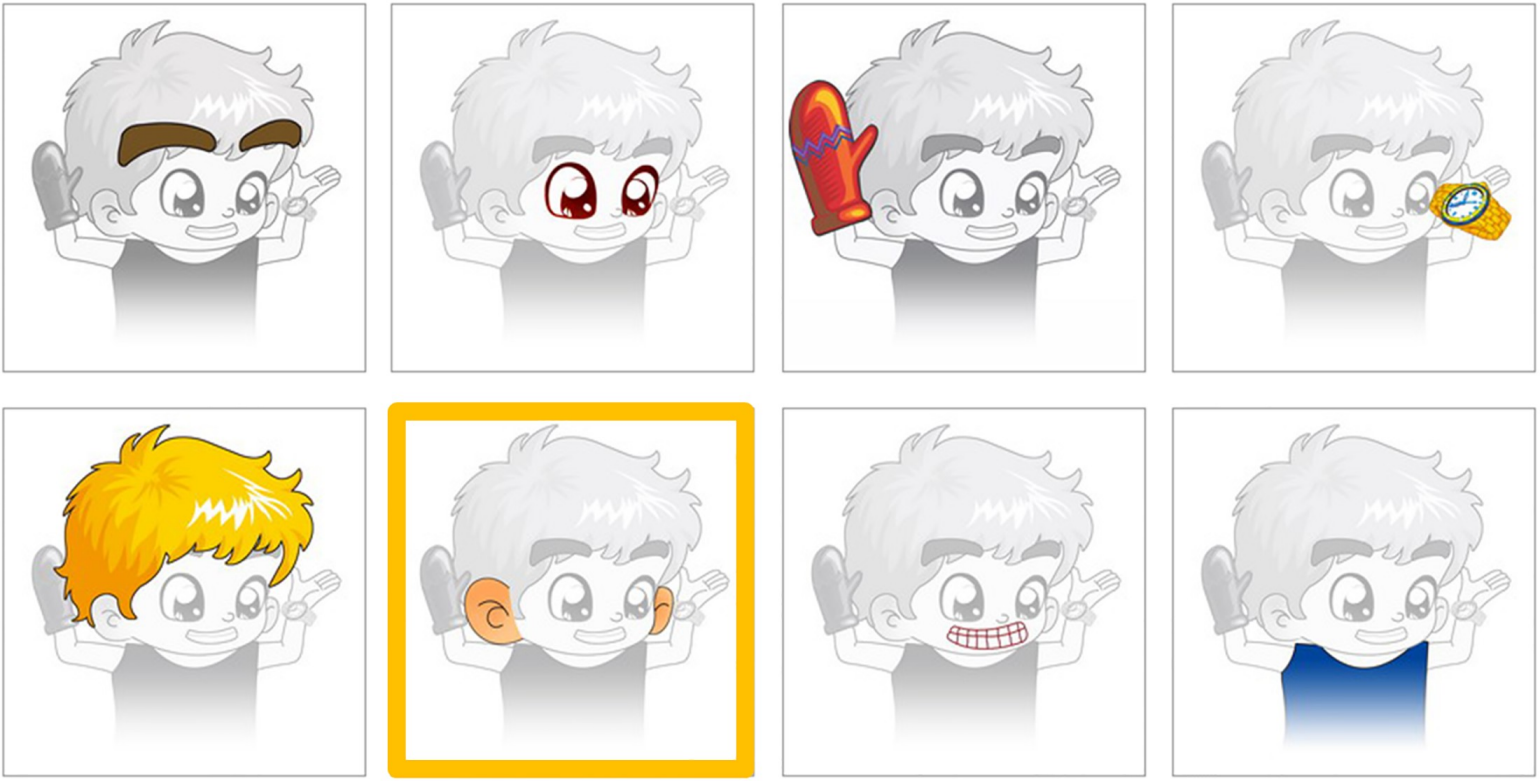
A sample screen from the *“body parts & clothing items”* testing set. After the spoken word was presented via the loudspeaker and the participant responded by touching one of eight pictures on the screen, a yellow frame surrounding the selected picture would blink to highlight the selection; in this sample, the word was /jɛnˇ tɕɪŋˉ/, meaning *“eyes”* and corresponding to the Chinese characters*“眼睛”*.

In a sound-treated environment, the test materials were presented using a custom software installed in a PC via an E-MU 0404 USB 2.0 Audio/ MIDI Interface and two loudspeakers. The presentation level was calibrated at 65 dB SPL via two loudspeakers placed one meter away from the participant; one loudspeaker was set at a 0-degree azimuth, while the other was set at a 90- or 270- degrees azimuth by random selection relative to the participant’s position. To present each test item, pictures representing all eight items in the respective test set were randomly assigned to appear in a display format of a four-columns to two-rows matrix on a touch screen monitor, therefore creating an eight-alternative forced choice (AFC) response setup. A sample screen from the *“body parts and clothing items”* test set is shown in Fig 1. After the eight pictures were shown, the test administrator pressed a hot key to play the test item when the participant was ready. The test item was then presented via the loudspeaker at a 0-degree azimuth. The participant was then required to touch the picture representing the test item from among the eight picture choices. No time limit was set. After the participant touched a picture, a yellow frame immediately flashed around the selection, which was then logged in the software for off-line analysis. Once a picture was selected, no revision was allowed. This was immediately followed by a display of the eight pictures corresponding to the next test item on the touch screen monitor. This procedure was repeated until all 24 test items had been presented, which constituted one round of testing. The 24 test items were presented in random order.

Familiarization trials were conducted in a quiet environment to familiarize the participants with the test items before initiating the testing rounds in a noisy environment. During the first familiarization trial round, if the participant scored any item incorrectly, the administrator then indicated the correct response to train the participant. In the second familiarization trial, such feedback and training were not provided. All but five participants received a score of 100%; the remainder received a score of 95.8% (23 of 24 correct). The two familiarization trials took around 10–15 minutes to complete. All 40 subjects then proceed to the formal testing-in-noise trials.

The first testing-in-noise trial began at a -9dB SNR, with the fixed noise level of 65 dB SPL and test item level of 56 dB SPL. If a subject received a score of at least 75% (18 of 24 correct) in this initial test trial, the SNR was reduced in subsequent test trials such that the intensity level of the test items was reduced in 3-dB steps until the score decreased to 25% (6 of 24 correct) or below. Alternately, if the first testing-in-noise trial at -9 dB SNR yielded a score below 75%, the SNR was increased in subsequent test trials (i.e., the intensity of test items increased, in 3-dB steps) until the score reached at least 75%. Subsequently, the SNR was again decreased by 3-dB steps in subsequent test trials as previously described. This procedure aimed to capture the range of performance-SNR (P-SNR) function correct scores from 75% to 25% among individual participants. As the 24 test items were presented in an eight-AFC closed set test format and the participants were trained to recognize the test items and their corresponding pictures during the testing trials in a quiet setting, the effects of the participants’ linguistic knowledge and memory of the test materials across the test trials were assumed to be equal. Although no feedback was provided on response accuracy, the participants received occasional reinforcement on their effort and attention.

There were two noise-direction conditions for the testing-in-noise trials: Speech Front/Noise Side (NS), i.e. the spatially separated condition, and Speech Front/Noise Front (NF)., i.e. the spatially co-located condition. In the NS condition, the test items were presented from the loudspeaker at a 0-degree azimuth, and noise was played from the loudspeaker at either a 90- or 270- degrees azimuth (randomly selected for each participant). In the NF condition, the test items and noise were both presented from a 0-degree azimuth. Under both the NS and NF conditions, the noise was always initiated one sec before the test item and continued to play until 0.5 sec after the test item concluded. The participants were all tested first in the NS condition and then in the NF condition during the same half-day session. This fixed testing condition arrangement aimed to demonstrate that NF was a more difficult testing condition, even though the participants might have gained some practice effects from the previously administered NS condition. To complete the P-SNR functions specified above, three test trials were required for 24 and 22 participants; four test trials for 12 and 10 participants; and five test trials for 4 and 8 participants, for NF and NS respectively.

The P-SNR psychometric functions were obtained according to the mathematical procedures described by Nissen and colleagues [29]. The logistic regression intercept (a) and regression slope (b) values were obtained based on the proportion of correct scores (p) collected at each SNR presentation level, as shown in Eq (1). The calculations were conducted separately for individual participants and for individual test items under the NS and NF conditions.

The regression intercept (a) and regression slope (b) values obtained from Eq (1) were then fitted into Eq (2). The P-SNR psychometric functions were then created by generating the percentage of correct scores (P) at different SNR levels in 1-dB steps. Eq (1) was then solved to Eq (3) and simplified to Eq (4) to derive the SNR for a 50% correct score (SNR-50%), using the regression intercept (a) and regression slope (b) values obtained from Eq (1).

The SNR-50% allows comparisons among all test results located at the midpoint of the percentage scale, where scores exhibit the maximum variability and therefore exhibit greater inter-score homogeneity. Compared with percentage scoring, the SNR-50% is a more reliable comparative measure of scores obtained under different test conditions because it prevents the comparison of scores that fall in different regions of the percentage scale and thus exhibit heterogeneous variability (e.g., scores that fall in the lower and upper boundaries of the percentage scale have much lower variability than those near the midpoint) [30–33].

The SRM was calculated as the improvement (if any) of the SNR-50% from NF to NS, as shown in Eq (5).

$$\text{log}\frac{p}{1-p}=a+b\times SNR$$ (1)

$$P=(\frac{\text{exp}(a+b\times SNR)}{1+\text{exp}(a+b\times SNR)})\times 100\%$$ (2)

$$SNR=\frac{\text{log}\frac{p}{1-p}-a}{b}$$ (3)

$$If\;p=0.5,\;SNR-50\%=\frac{-a}{b}$$ (4)

$$SRM=SNR-50{\%}_{NF}-SNR-50{\%}_{NS}{}_{}$$ (5)

The logistic regression results for individual participants revealed that the P-SNR function was significant (p < .05) in nearly all subjects under both the NS and NF conditions, except for two subjects (S9 and S18) whose functions were not significant (p > .05) in the NS condition. All data from those two subjects were removed from future analyses.

Similarly, the logistic regression results for the individual test items showed that the P-SNR functions for most of the 24 test items were significant (p < .05). However, five and four items in the NF and NS conditions, respectively, did not yield significant logistic regression results (p > .05). The same four items with insignificant regression results, *“Ear”*, *“Hair”*, *“Cloth” and “Butterfly”*, were observed in both the NS and NF conditions, while *“Watch”* yielded insignificant regression results only in the NF condition. To maintain the same set of test items to be used in the adaptive testing for both NF and NS conditions in Experiment 2, all data collected for these five test items were removed from both NF and NS, and therefore only the data of the remaining 19 test items were included in future analyses.

## Results

Table 2 presents the SNR-50% scores obtained for the 19 test items that yielded significant logical regression results under both the NF and NS conditions. The SNR-50% scores ranged from -19.16 dB to -9.35 dB for the NF condition (mean (M) = -13.34 dB, standard deviation (SD) = 2.40 dB) and -13.76 dB to -22.45 dB for the NS condition (M = -17.11 dB, SD = 2.63 dB). Test items that received more negative SNR-50% scores were easier to recognize, as less favorable (i.e. more negative) SNRs yielded the 50% score, whereas test items with less negative SNR-50% scores were more difficult to recognize because more favorable SNRs (i.e. more positive) were needed to yield the same 50% score. The test items were therefore by no means homogenous in terms of difficulty. The heterogenous P-SNR functions across the test items could be due to differences in lexical neighborhood frequency, phonotactic structure and lexical access of the target words in the test items [34]; and differences in the interaction of the same noise but different test items, i.e. differences in the instantaneous SNR changes in different frequency regions along the presentation timeline of the test items.

**Table 2:** Signal-to-noise ratio for 50% correct scores (SNR-50%, dB SNR) obtained from the Speech Front/Noise Side (NS) and Speech Front/Noise Front conditions (NF) and the spatial release from masking (SRM) values yielded from the two conditions.

Set	Test item	SNR-50% (dB SNR)		SRM (dB)	Regression intercept (*a*)		Regression slope (*b*)		Intensity adjustment to reach mean SNR-50% (dB)	
		NF	NS		NF	NS	NF	NS	NF	NS
*Animals*	Rabbit	-19.16	-21.81	2.65	5.46	3.97	0.29	0.18	-5.82	-4.70
	Elephant	-16.76	-21.22	4.46	13.26	13.75	0.79	0.65	-3.42	-4.11
	Rooster	-11.44	-14.13	2.69	2.72	3.21	0.24	0.23	1.89	2.98
	Butterfly	-	-	-	-	-	-	-	-	-
	Tiger	-13.72	-15.16	1.44	6.01	2.97	0.44	0.20	-0.38	1.95
	Frog	-15.35	-22.45	7.10	8.10	6.69	0.53	0.30	-2.01	-5.35
	Dog	-12.56	-13.95	1.40	3.64	3.47	0.29	0.25	0.78	3.15
	Panda	-14.66	-16.17	1.50	7.52	4.79	0.51	0.30	-1.33	0.94
*Body parts and clothing items*	Ear	-	-	-	-	-	-	-	-	-
	Eyebrow	-9.35	-14.36	5.01	2.70	4.31	0.29	0.30	3.98	2.75
	Glove	-14.44	-17.57	3.14	8.37	7.19	0.58	0.41	-1.10	-0.47
	Cloth	-	-	-	-	-	-	-	-	-
	Hair	-	-	-	-	-	-	-	-	-
	Teeth	-12.59	-17.21	4.63	7.31	6.01	0.58	0.35	0.75	-0.11
	Eye	-11.47	-16.96	5.49	4.70	6.65	0.41	0.39	1.86	0.14
	Watch	-	-	-	-	-	-	-	-	-
*Everyday objects*	Television	-10.00	-15.70	5.70	5.74	7.26	0.57	0.46	3.33	1.40
	Towel	-13.22	-15.32	2.10	5.71	4.50	0.43	0.29	0.11	1.79
	School Bag	-14.82	-17.84	3.02	3.51	3.37	0.24	0.19	-1.48	-0.74
	Cup	-12.60	-16.95	4.35	7.13	6.93	0.57	0.41	0.74	0.16
	Eyeglasses	-11.22	-17.53	6.31	8.29	5.79	0.74	0.33	2.11	-0.42
	Key	-14.79	-16.84	2.05	9.76	8.05	0.66	0.48	-1.45	0.27
	Toothbrush	-14.29	-20.08	5.79	10.10	3.80	0.71	0.19	-0.95	-2.97
	Umbrella	-10.94	-13.76	2.82	4.85	4.14	0.44	0.30	2.40	3.35
	*M*	-13.34	-17.11	3.77	6.57	5.62	0.49	0.33	*1.89	*1.99
	*SD*	2.40	2.63	1.79	2.74	2.53	0.17	0.12	*1.42	*1.67

* Means (M) and standard deviations (SD) were calculated based on absolute values, regardless of the direction of intensity adjustment.

To develop an adaptive testing procedure, these 19 test items needed to be adjusted to the same difficulty level. Using the mean SNR-50% as a reference point, the intensity level of an item with an SNR-50% score more negative (i.e., easier) than the reference point was reduced by the difference between the SNR-50% score of the specific item and the mean SNR-50%. For example, the first test item, *“Rabbit”*, received a SNR-50% score (-19.16 dB) more negative (or easier) than the mean SNR-50% score (-13.34 dB) under the NF condition (Table 2). Therefore, the intensity of this test item was reduced by 5.82 dB (-19.16 dB minus -13.34 dB) to align it with the mean difficulty level. Using this logic, for an item with a less negative SNR-50% score (i.e., more difficult) than the reference point (mean SNR-50% score), the intensity level was increased accordingly. The direction (reducing or increasing) and amount (dB) of intensity adjustment for each item under the NF and NS conditions are shown in Table 2. The 19 test items were adjusted separately for the NF and NS conditions. Regardless of the adjustment direction, the mean intensity adjustments were 1.89 dB and 1.99 dB for the NF and NS conditions, respectively. After the intensity adjustments, the difficulty levels of the test items were aligned to the mean difficulty levels (thereafter designated the MD level) of -13.34 dB and -17.11 dB for the NS and NF conditions, respectively. Therefore, the MD level indicates the difficulty of the items under variable absolute intensity levels among the test items. This difficulty alignment procedure was required to develop the adaptive testing procedure in Experiment 2.

## Experiment 2—Development of the adaptive testing procedure

### Participants

Twelve children (6 boys, 6 girls) aged 4.83–5.25 years (M = 4.97 years, SD = 0.29 years) were recruited in Beijing. The youngest participant in Experiment 2 (4.83 years) was older than the one in Experiment 1 (3.5 years). It was attempted to recruit participants as young as 3.5 years for Experiment 2 but no participant at that age was available during the short two-week participant recruitment and testing period. All participants were native speakers of Mandarin. None of the recruited children had any reported problems with speech, language, hearing or cognitive development. Written consent was sought from the parents or guardians for the involvement of each participant in the study. This study was approved by the Human Research Ethics Committee of The Education University of Hong Kong. All participants underwent pure tone air-conduction play or standard audiometry at octave frequencies of 250, 500, 1000, 2000, 4000 and 8000 Hz. All participants were found to have normal hearing sensitivities with pure tone air condition thresholds of 15 dB HL or better in both ears at all test frequencies.

### Methods

Participants went through the same two familiarization trials in quiet as previously described in Experiment 1. All participants scored 100% in the second familiarization trial. Therefore, they were all allowed to proceed to the testing-in-noise adaptive procedure. In this testing-in-noise adaptive procedure, the same speech-spectrum weighted noise in Experiment 1 was used and its presentation level was always fixed at 65 dB SPL. The speech stimuli were presented after the noise with an onset delay of 0.5 s. The presentation order of the 19 items was randomized for each testing condition (NS and NF), and the first randomly selected item was then played at the MD level (-13.34 dB SNR for NF; -17.11 dB SNR for NS). Recall from Experiment 1 that five of the 19 tests items (one item from the *“animals”* test set and four items from the *“body parts and clothing items”* test set) had been excluded for further use in Experiment 2. Nevertheless, the same original eight pictures from each test set including those pictures of the five excluded test items were still displayed on the touch screen monitor although those five test items were never presented in the adaptive testing procedure. Therefore, the eight-AFC response setup was maintained among all test items in Experiment 2.

**Fig 2:**
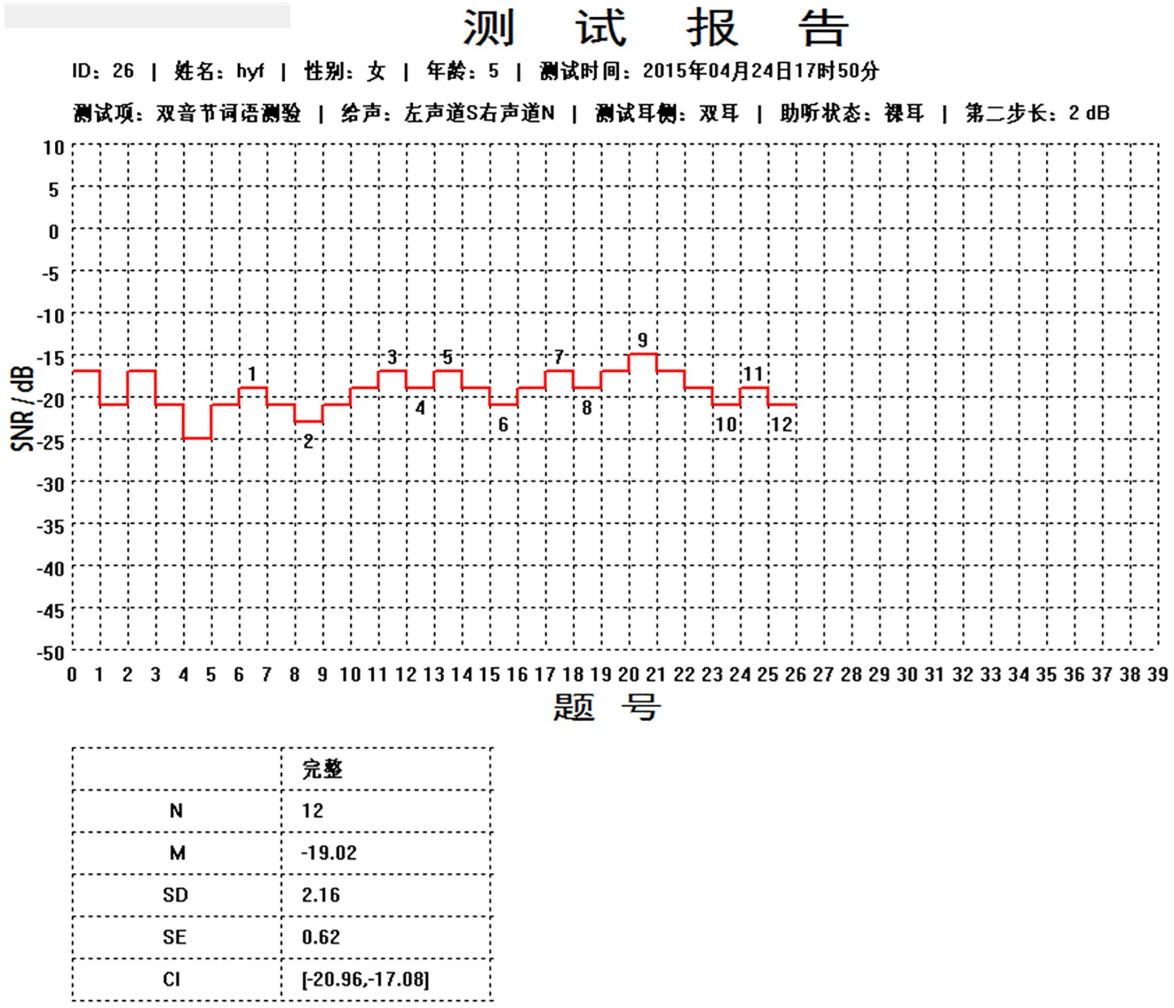
Display of a sample adaptive test result from the *MAPID-A* computer testing interface.

The adaptive testing procedures were applied using the following steps (1) to (12):

1. If the participant initially responded correctly to the first test item at the MD level (in dB SNR), the testing would proceed to step (3); otherwise the testing would proceed to step (2).
2. If the participant could not recognize the first test item, it was repeated, with the SNR increased in 4 dB steps, until the item was correctly identified. Testing then proceeded to step (3).
3. The item presentation SNR level was then decreased by 4 dB SNR for presenting the second test item.
4. A correct recognition of the second test item warranted a 4-dB SNR decrement in the presentation SNR level of the third item, whereas an incorrect recognition of that item warranted a 4-dB SNR increment in the presentation SNR level of the third item.
5. Step (4) was repeated until the first 6 items were presented. Therefore, after the first five test items were presented, the presentation SNR level step size adjustment (increment or decrement) was 4 dB SNR.
6. After the sixth item was presented, the step size was reduced from 4- to 2- dB SNR for the remainder of the adaptive testing procedure. In other words, the correct or incorrect recognition of the sixth test item warranted a 2-dB SNR decrement (raise difficulty) or increment (lower difficulty), respectively, in the presentation SNR level of the seventh item. The presentation SNR level (in dB SNR) of the sixth item marked the starting point of the first excursion.
7. The adaptive testing procedure continued to run as described in step 6 for the next item. The reversal point where the presentation SNR level changed direction (from decrement to increment or vice versa) marked the endpoint of the first excursion. For example, Fig 2 presents the screen display of an adaptive test result wherein the sixth item was presented at a -21.11 dB SNR (i.e., starting point of the first excursion) and an incorrect recognition response led to a 2-dB SNR increment in the presentation intensity level of the seventh item to -19.11 dB SNR. The correct recognition of the seventh item led to a change in the presentation SNR level direction (i.e. from increment to decrement) when the eighth item was played at -21.11 dB SNR. Therefore, the play level of the seventh item at -19.11 dB SNR marked the endpoint of the first excursion. As this was the reversal point, it also marked the starting point of the second excursion. In summary, the first excursion ran from -21.11 to -19.11dB SNR, with a midpoint of -20.11 dB SNR.
8. Similarly, as shown in Fig 2, the presentation SNR level of the seventh item at -19.11 dB SNR was the starting point of the second excursion. Due to the correct recognition of both the seventh and eighth items, the eighth and ninth items were presented at -21.11 and -23.11 dB SNR, respectively, after successive 2-dB SNR decrements. However, incorrect recognition of the ninth item caused a change in presentation SNR level direction again (i.e., from decrement to increment) in the 10th item, which was presented at -21.11 dB SNR. Therefore, the presentation SNR level of the ninth item at -23.11 dB SNR marked the end of the second excursion and the starting point of the third excursion. The second excursion ran from -19.11 to -23.11dB SNR, with a midpoint of -21.11 dB SNR. Adaptive testing continued until 12 excursions were completed.
9. If all 19 items were presented in randomized order before the 12 excursions were completed, a second round of 19 items in another randomized order were presented continually until all 12 excursions were completed. As in Fig 2, 26 items were played to yield the 12 excursions. Therefore, seven items (26 minus 19) were repeated in the second round to complete the testing for 12 excursions.
10. The midpoints of the 12 excursions (in dB SNR) obtained from the presentation of the sixth to the final item were averaged to yield the M and SD of the adaptive SNR-50% score (aSNR-50%).
11. The standard error (*SE*) was calculated using the formula (*SE* = *SD*/√*N*), where *N* = 12 or the total number of excursions.
12. The 99% confidence interval (*CI*) was calculated using the formula: (*M*–(*t* x *SE*)) to (*M* + (*t* x *SE*)), where *t* is the t-value of 3.106 (two-tailed test) for a degree of freedom of 11 (for *N* = 12).

Three test conditions were applied; of these, two conditions were spatially separated–Speech Front/Noise Right (R) and Speech Front/Noise Left (L)–and one condition was spatially co-located—Speech Front/ Noise Front (F). In the spatially separated conditions, the test items were played from a loudspeaker at a 0-degree azimuth, and noise was played from a loudspeaker at a 90- or 270-degrees azimuth in the R and L conditions, respectively. In the spatially co-located condition (F), the test items and noise were both played from the same loudspeaker at a 0-degree azimuth.

One boy and one girl were randomly assigned to one of six possible test condition sequences of the three test conditions: R-L-F, R-F-L, L-R-F, L-F-R, F-R-L and F-L-R. After a test condition (e.g., R-L-F) was completed, the participants were retested using the same three conditions in the reverse test condition sequence (e.g., F-L-R) to counterbalance the test sequence for each participant. Therefore, each participant underwent two rounds of testing for the same test condition.

## Results

Table 3 shows the individual participants’ aSNR-50% scores and SDs for each test condition in the original and reverse test sequences. The two test results from the same test condition were combined using the formulae from [35] to generate the combined N, M and SD values (see Table 4).

**Table 3:** The adaptive signal-to-noise ratio for 50% correct score (aSNR-50%, dB SNR) and spatial release from masking (SRM; dB) of individual participants.

Participant	^+^ Test sequence	aSNR-50%									SRM (dB)			
		Noise Left (L)			Noise Right (R)			Noise Front (F)			SRM (L vs. F)		SRM (R vs. F)	
		*M*	Combined *M*	Combined *SD*	*M*	Combined *M*	Combined *SD*	*M*	Combined M	Combined *SD*	-	Combined	-	Combined
F1	RFL	-17.38	-19.07	3.83	-11.93	-14.02	3.06	-11.61	-11.02	2.56	5.77	8.05	0.32	3.00
	LFR	-20.75			-16.11			-10.43			10.32		5.68	
M1	FRL	-14.75	-17.71	3.92	-16.75	-17.34	2.65	-11.89	-12.34	2.69	2.86	5.37	4.86	5.00
	LRF	-20.66			-17.93			-12.79			7.87		5.14	
M2	RFL	-16.66	-17.66	3.19	-19.11	-19.20	3.20	-11.79	-11.20	2.85	4.87	6.46	7.32	8.00
	LFR	-18.66			-19.29			-10.61			8.05		8.68	
F2	LFR	-18.66	-18.52	1.92	-18.2	-17.79	2.50	-11.43	-12.80	2.85	7.23	5.73	6.77	5.00
	RFL	-18.38			-17.38			-14.16			4.22		3.22	
M3	LRF	-17.84	-17.25	2.37	-18.56	-15.79	3.77	-10.98	-10.98	2.06	6.86	6.27	7.58	4.81
	FRL	-16.66			-13.02			-10.98			5.68		2.04	
M4	FLR	-19.29	-18.38	2.30	-21.66	-23.43	2.87	-12.07	-11.57	3.05	7.22	6.81	9.59	11.86
	RLF	-17.47			-25.2			-11.07			6.4		14.13	
M5	LFR	-17.56	-18.56	2.66	-21.75	-21.52	2.59	-12.52	-13.11	2.63	5.04	5.45	9.23	8.41
	RFL	-19.56			-21.29			-13.7			5.86		7.59	
M6	RLF	-19.84	-20.48	2.32	-18.56	*	*	-11.16	-11.71	2.46	8.68	8.77	7.4	*
	FLR	-21.11			*			-12.25			8.86		*	
F3	FLR	-15.11	-15.98	3.11	-11.84	-11.70	2.84	-9.79	-10.93	2.85	5.32	5.05	2.05	0.77
	RLF	-16.84			-11.56			-12.07			4.77		-0.51	
F4	RLF	-17.56	-18.25	3.59	-24.38	-23.07	3.17	-11.07	-11.93	2.23	6.49	6.32	13.31	11.14
	FLR	-18.93			-21.75			-12.79			6.14		8.96	
F5	FRL	-18.84	*	*	-19.84	*	*	-10.98	*	*	7.86	*	8.86	*
	LRF	*			*			*			*		*	
F6	LRF	-18.02	-18.52	2.84	-18.93	-19.2	2.45	-10.25	-9.52	2.47	7.77	9.00	8.68	9.68
	FRL	-19.02			-19.47			-8.79			10.23		10.68	
	**Mean**	-18.24	-18.21	2.91	-18.39	-18.31	2.91	-11.53	-11.55	2.61	6.71	6.66	6.89	6.77
	***SD***	1.64	1.09	0.67	3.77	3.81	0.40	1.22	1.00	0.29	1.86	1.53	3.80	4.23

^+^Test sequence: R, Speech Front/Noise Right; L, Speech Front/Noise Left; F, Speech Front/ Noise Front.

*Missing data.

**Table 4:** Formulae used to calculate the combined *N*, *M* and *SD* from the two test rounds of the same condition in each participant [35].

	Test Round		Combined First & Second Test Round
	First	Second	
Sample size	*N*_*1*_	*N*_*2*_	*N*_12_ = *N*_1_ + *N*_2_ = 24
Mean	*M*_*1*_	*M*_*2*_	$M_{12}=\frac{N_{1}M_{1}+N_{2}M_{2}}{N_{1}+N_{2}}$
Standard Deviation	*SD*_*1*_	*SD*_*2*_	$SD_{12}=\sqrt{\frac{\left(N_{1}-1\right)SD^{2}+\left(N_{2}-1\right)SD_{2}^{2}+\frac{N_{1}N_{2}}{N_{1}+N_{2}}\left(M_{1}^{2}+M_{2}^{2}-2M_{1}M_{2}\right)}{N_{1}+N_{2}-1}}$
Standard error	*SE*_*1*_	*SE*_*2*_	$SE_{12}=SD_{12}\sqrt{N_{12}}$

The group average aSNR-50% scores for the F, R and L conditions were -11.55, -18.31 and -18.21 dB SNR, respectively. The aSNR-50% scores obtained from the R and L conditions significantly outperformed those from the F condition, with the following comparisons: R outperformed F, *t*(11) = 7.23, *p* < .000005 and L outperformed F, *t*(11) = 17.31, *p* < .0000001. The aSNR-50% scores did not differ significantly between the R and L conditions (*t*(11) = -0.21, *p* = .84).

The mean SRMs for the right and left ears were 6.77 dB SNR (*SD* = 4.23 dB SNR) and 6.66 dB SNR (*SD* = 1.53 dB SNR), respectively. The SRM values obtained from the two ears were not significantly different (*t*(11) = 0.21, *p* = .84).

Table 5 presents the distributions of the 99% *CI*s of the mean aSNR-50% scores obtained from the 12 participants. Recall that each participant underwent two rounds of testing per test condition. Therefore, the combined results of two trials are shown. One participant did not complete the retesting for the R test condition, and another did not complete the retesting for all test conditions. One-way analysis of variance (*ANOVA*) revealed no significant main effect of the test conditions (L, R and F) on the 99% *CI* of the mean aSNR-50% scores (*F* (2,42) = 0.64, *p* = 0.53), and a post-hoc Tukey Honest Significant Difference (*HSD*) test revealed no difference in any of the pairwise comparisons among the three conditions. Therefore, all individual subject results obtained under different test conditions were collapsed to yield an overall mean 99% MAPID-ue of ±1.61 dB SNR. A stringent criteria of non-overlapping 99% *CI*s of the mean aSNR-50% was adopted to demarcate significant different results between test conditions. In other words, two times the value of 1.61 dB SNR, or 3.22 dB SNR, was the average critical difference required to confirm that the scores obtained under two test conditions differed significantly for the same participant. However, the actual critical difference for an individual participant would depend on the distribution of the midpoints of the 12 excursions and, hence, the aSNR-50% obtained from the participant’s own test results. The SD of the average critical difference value of 0.56 dB SNR (two times 0.28 dB) revealed a narrow distribution of the critical differences among the participants.

**Table 5:** Means, standard deviations (*SD*s) and range of distribution (99% confidence interval; *CI*) of the mean adaptive signal-to-noise ratio for 50% correct score (aSNR-50%; dB SNR) obtained from 12 participants under each test condition (combined results of two test rounds).

	99% *CI* of mean aSNR-50% (dB SNR) Test condition			
	L (*n* = 11)	R (*n* = 10)	F (*n* = 11)	Overall (*N* = 32)
Mean	±1.67	±1.67	±1.49	±1.61
*SD*	±0.38	±0.23	±0.17	±0.28
Max	±2.25	±1.41	±1.75	±2.25
Min	±1.10	±2.16	±1.18	±1.10

The 99% *CI* of the mean aSNR-50% was calculated by combining data from the 24 excursions in the two test rounds and obtained from each participant under the three noise direction test conditions. Those three test conditions were compared among themselves using pairwise comparisons (i.e., L vs. F, R vs. F and L vs. R) to investigate whether a statistically significant SRM could be obtained within each participant. If the 99% *CI*s of the mean aSNR-50% scores of the two test conditions were non-overlapping, the comparison was deemed statistically different. Table 6 presents the number of participants with a statistically significant SRM (e.g., L outperformed F or R outperformed F).

**Table 6:** Results of an within-participant pairwise comparison among the three noise testing conditions.

Results of pairwise comparison among the three test conditions	Total number of participants with statistically different results/total number of participants who completed all testing in the pairwise comparison (%)*
R outperformed F (SRM)	9/10 (90%)
L outperformed F (SRM)	11/11 (100%)
F outperformed R	0/10 (0%)
F outperformed L	0/11 (0%)
L outperformed R (Left ear advantage)	2/10 (20%)
R outperformed L (Right ear advantage)	3/ 10 (30%)

*One participant did not complete testing for all three conditions, and another participant did not complete testing for the R condition. Therefore, the total number of participants was 10 or 11.

SRM, spatial release from masking

The within-participant performance reliability was assessed based on the results of the two test rounds for the same noise testing condition. If any overlapping regions (albeit small) were found between the two 99% *CI*s from the two test rounds, obtained from the two aSNR-50% scores yielded separately from those two test rounds, the results of those two test rounds were deemed non-significantly different or, in other words, reliable. The within-participant reliability results obtained from the completed data sets of 10, 11 and 10 participants under the L, R and F conditions were 90.1%, 80% and 100%, respectively. The overall reliability was 90.6%.

The average time needed to complete one adaptive test trial per noise condition and per participant was 4.2 min (SD = 2.2 min). In other words, around 84% of the participants completed a test trial within 6.5 min.

## Discussion

### *MAPID-A* is a powerful and efficient clinical tool for measuring speech-recognition in noise and SRM

This study was conducted to develop a reliable measure of speech-recognition-in-noise for young children—the *MAndarin spoken word—Picture IDentification test in noise—Adaptive (MAPID-A)*—that would especially allow within-participant statistical comparison across different test conditions. To the authors’ knowledge, such a tool is not readily available in the literature. The *MAPID-A* would be clinically invaluable for assessing the maturational time course of speech-recognition-in-noise among individual young children, using their own performances as the baseline. This within-participant comparison procedure has the power to delineate any statistical differences between two test conditions or among multiple test conditions. An average aSNR90% 99% *CI* of ±1.61 dB SNR or a non-overlapping critical difference of 3.22 dB SNR is a very stringent criterion for delineating a statistical difference. The adaptation of this stringent criterion endowed the *MAPID-A* with the power to detect subtle changes in speech-recognition-in noise and, as shown in this study, to detect SRM in young children. Overall, 90% and 100% of the participants gave significantly better aSNR-50% performances in the NR and NL conditions, respectively, than in the NF condition. The *MAPID-A* adopted a closed-test picture-point testing format initiated by the presentation of a spoken-word. This testing procedure is feasible and practical for the testing of children as young as 4–5 years of age. Given the narrow *CI*s obtained from the SNR-50% measures in young children with normal hearing, there is a potential to apply the *MAPID-A* to test young children with hearing impairments. The testing results would help reveal if there is any statistically significant within-participant speech-in-noise recognition changes between monaural versus binaural hearing prosthesis (hearing aids, middle ear implants, cochlear implants, etc.) fittings, monaural cochlear implant versus bimodal fittings (hearing aid and cochlear implant fitted in contralateral ears), or monoaural versus binaural cochlear implant fittings. Apart from comparing the monaural versus binaural aided hearing mentioned above, the *MAPID-A* can also be employed to detect if there are any statistically significant speech-recognition-in-noise improvements with the introduction of new pre-processing noise reduction algorithm, sound coding strategy or other software upgrades in the same hearing prosthesis.

### SRM in children

As described for Experiment 2, the mean aSNR-50% values obtained from the combined results for the F, R and L conditions were -11.51 dB, -18.45 dB and -18.27 dB, respectively, and the mean SRMs for the R vs. F and L vs. F conditions were 6.66 dB (*SD* = 1.53 dB) and 6.77 dB (*SD* = 4.23 dB), respectively. The results are comparable to those obtained by Yuen and Yuan [6], who reported average SNR-50% values of -14.58 and -20.37 dB SNR for the NF and NS conditions, respectively, and a mean SRM of 5.76 dB. The differences were attributed to the following reasons. First, although all test materials used in this study and in the study by Yuen and Yuan [6] were identical, this study used a new set of recordings (albeit still using a female voice). Second, the pictures used in the *“animals”* and *“everyday objects”* test sets were changed from the cartoon pictures used in the *MAPPID-N* to photos in this version of *MAPID-A*. Third, the mean SNR-50% reported by Yuen and Yuan [6] was obtained from a logistic regression of the P-SNR functions, whereas the aSNR-50% was obtained using an adaptive testing procedure. Previous studies of children with normal hearing yielded an SRM of 5.25 dB [36], 7.7–10.0 dB [37], approximately 4.5–6.8 dB [38], 3.0–4.3 dB [21], 2.8–3.5 dB [39], 6 dB [40], approximately 5 dB [41] and 5.5–6.4 dB [42]. The magnitudes of the SRMs attained in this study (6.66–6.77 dB) were near the midrange of those previous pediatric studies (2.8–10.5 dB). Most of the aforementioned studies on SRM for children: a) maintained the same type of signal used in the speech (e.g., speech signal from the same male voice) and in the noise (e.g., speech weighted noise with the same frequency spectrum, or speech babble with the same speakers) testing conditions; and b) used different type of signals for the speech signal and the noise signal, across the spatially co-located and the spatially separated test condition. However, Cameron and colleagues [43] measured SRM differently as spatial advantage and total advantage. Spatial advantage was defined as the improvement of speech reception threshold (SRT) from a speech-noise spatially co-located testing condition to a spatially-separated condition when the same speaker’s voice was used in both the speech and noise. Total advantage was defined as the improvement from speech-noise spatially co-located testing condition above (in spatial advantage which uses the same speaker’s voice) to a different spatially-separated condition where different speakers’ voices were used in the noise signal. Cameron and colleagues [43] reported a spatial advantage SRM of 9.2–10.5 dB and total advantages SRM of 10.2–10.5 dB.

Differences in the SRM values across those studies may be attributed to differences in the testing procedures, calculation methods, age ranges of participants, speech materials (open-set vs. closed-set; sentences vs. words), masker (speech babble vs. stationary noise), required response type (word or picture identification vs. free recall), masker presentation direction in the spatially separated condition (one side only vs. both sides) and speaker delivering the speech babble masker (same vs. different from the speaker delivering target speech test materials).

From the summary of Yuen and Yuan [6] on the ten previous studies that investigated the development of spatial release from masking in children [6, 21, 36–41, 43, 44], only three studies—Garadat and Litovsky [37], Van Duen et al. [21] and Yuen and Yuan [6]—reported error estimates, i.e. SDs of the SRMs obtained from group results. However, none of those three studies reported error estimates from individual participant results probably due to limitations of the speech-recognition-in-noise testing procedures. To the author’s knowledge, the current study is the first pediatric study which offered within-participant error estimates of speech-recognition-in-noise performance, i.e. SNR-50%, from different noise directions. Please refer to Table 3 for individual participant’s SDs of SNR-50% in the three different noise direction conditions. Therefore, not just the degree of SRM, but the presence of SRM could be determined statistically within each young child participant. *MAPID-A* offers unprecedented value for future research and clinical practice to evaluate within-participant speech-recognition-in-noise and SRM changes for young children.

The SRM is calculated by subtracting the easier (i.e., lower aSNR-50%) speech-noise spatially separated condition from the more difficult (i.e., higher aSNR-50%) speech-noise spatially co-located condition as the baseline. The correction from the baseline condition focuses only on the degree of masking release of the noise source relative to the speech source between the spatially co-located and spatially separated conditions and disregards the absolute speech-in-noise performance level of the participants. This two-point data subtraction method, which uses the participant as her or his own control, minimizes possible interference from non-auditory confounding factors such as language competence, attention, working memory, instruction and task-related factors. We believe that SRM can be potentially used as a non-language-specific auditory processing measure reflective of the performance of auditory stream segregation in spatial hearing. Further cross-language cohort studies using similar testing materials (e.g., disyllabic words suitable for preschool children) and the same experimental procedures would help to determine whether the degree of SRM is consistent across children at the same age who speak different native languages.

### SRM as an outcome measure of the neurophysiological development of the auditory neural pathway for speech-recognition-in-noise

As noted in the Introduction, the SRM is a collective measure of an individual’s speech-recognition-in-noise influenced by head shadow and binaural unmasking. Regarding head shadow, under the spatially separated condition, the interaural time, level and frequency spectrum asymmetries of the incoming speech and noise signals depend on the physical size of the listener’s head and torso, as well as the positions of the ears. Among girls, the head circumference generally does not reach an adult level until 16 years of age, whereas boys do not achieve an adult head circumference until 21 years of age [45–47]. As infants and children have small heads than adults, the magnitude of their interaural cues, i.e. interaural time difference (ITD) and interaural intensity difference (IID), are smaller [48, 49]. Clifton and colleagues [48] estimated that the ITD between 0-degree and 90-degrees azimuth decreased from close to 700 microseconds in adults to around 400 microseconds in newborn infants. Fels and colleagues’ head-related transfer functions showed that IID at 4 kHz decreased from 20 dB from adults to around 10 dB in 6-month-old infants [49]. Therefore, the head shadow effect would be stronger as children’s heads grow which offers more salient interaural asymmetries and would yield an improved speech-recognition-in-noise as demonstrated by a better (i.e., smaller or more negative) aSNR-50% under the noise side condition. This would enable the higher level auditory neural system to compute the differences and exhibit the binaural unmasking effect, as discussed below. Whether a child would benefit maximally from the head shadow would also depend on whether he or she could selectively direct more attention to the masked target speech signal received in the ear with better SNR and less attention to the masked target speech signal received in the contralateral ear with a lower SNR.

Regarding binaural unmasking, the auditory neural system of an older child, compared with that of a younger peer, would be more robust in detecting interaural differences (albeit subtle ones) when decorrelating the noise from the speech to enable better speech recognition. The auditory neural system continues to develop throughout childhood and until early adolescence (and possibly beyond). The levels of the medial and lateral superior olives (MSO and LSO, respectively), inferior colliculus, lateral lemniscus, thalamus and auditory cortex are anatomical landmarks where the ipsilateral and contralateral auditory pathways meet, which allows the masked signals received by these two pathways to be compared and interpreted [50]. The neuronal discharge patterns at the MSO and LSO vary according to the extent of the interaural asymmetries over time and the intensity and correlation of the two copies of the signal received from the two pathways. If an externally applied interaural acoustic delay offsets the internal interaural transmission delay, neurons in the MSO discharge maximally (excitation) whereas those in the LSO discharge minimally (inhibition). These complementary excitation-inhibition patterns at the MSO and LSO are believed to be neural collates of the primary stages of binaural unmasking and SRM. Those precise neural firing patterns at the brainstem and higher regions require a mature and experienced auditory neural system. Spatial sound localization is refined by experience. Animal studies have shown that the central auditory pathway continually exhibits plastic changes in the processing of monaural and binaural sound streams at the level of the superior colliculus [51]. Identification of the target sound stream requires an auditory memory of sound patterns consequent to auditory experiences involving the auditory cortex [52].

Some previous studies have suggested that the auditory neural system of a 3–4-year-old child has already achieved an adult-level SRM performance [37, 39–41, 44]. However, given the complex and precise computations required for binaural unmasking and SRM, there is every reason to suspect that the SRM would continue to increase beyond early childhood and would not reach an adult level until children reach their late childhood and adolescent years. Indeed, a number of studies support the late developmental completion of SRM beyond early childhood [6, 21, 38, 42, 43]. In future studies, the development of efficient and reliable clinical tools, like the *MAPID-A*, in other languages and regions would help to confirm the late developmental completion of SRM.

Apart from the efferent auditory neural system described above, the afferent auditory neural system, particularly the olivocochlear bundle (OCB), was found to affect hearing in the context of background noise [26, 53, 54]. Human studies have revealed that the inhibitory function of the medial OCB is strongly associated with the enhancement, or “antimasking”, of auditory nerve responses during the detection and recognition of target signals in noise [55–57]. The *MAPID-A* is a valuable tool to further explore behaviorally the acquired “antimasking” effect on speech-recognition-in-noise which has not been explored thoroughly in young children, given the unavailability of a reliable speech-recognition-in-noise measure for this population.

The ability of the *MAPID-A* to identify the presence of a statistically significant ear advantage from an individual participant’s results (rather than from group results) can reveal the participant’s functional asymmetry and cerebral dominance for speech-recognition-in-noise. A right ear advantage in the *MAPID-A* testing context indicates that the L condition outperforms the R condition, and the right ear was provided with a better SNR, compared to the left ear, and the vice versa for left ear advantage. From Table 6, a statistically significant ear advantage (i.e., non-overlapping 99% *CI*s of the mean aSNR-50% values in the L and R conditions) was observed for the right ear in three participants and the left ear in two participants, reflecting a significant functional asymmetry and cerebral dominance for speech-recognition-in-noise for these participants. Children with excessively weak right ear advantage or excessively strong left ear advantage were all found to have language-learning disorders [58], which was attributed to the right, instead of the left, hemisphere dominance for language. These children with language-learning disorders use the non-language specialized right hemisphere to process the temporally-based auditory-linguistic information, which is believed to be the cause of the poor development of auditory, phonologic and linguistic processing [59]. Individuals with language-learning disorders fail to inhibit the processing of the interfering right ear stimulus; they also fail to increase the activation of the right hemisphere due to a poor corpus callosum interhemispheric connection network, which makes it difficult to attend only to the left auditory space. The processing imbalance of the right and left cerebral hemispheres to the left and right auditory spaces, respectively, are the reasons why individuals with language-learning disorders fail to attend to important linguistic information selectively, especially in the presence of interfering signals from the contralateral processing channel [60], which is detrimental for auditory learning. The *MAPID-A* as a proven sensitive speech-recognition-in-noise measure would be a potentially valuable behavioral screening tool for young children with suspected communication disorders associated with atypical cerebral dominance for speech-recognition-in-noise such as auditory processing disorders and language-learning disorders, following validation in these clinical populations. Developmental norms for F, L, R, SRM, right ear and left ear advantage are urgently needed to screen those communication disorders associated with atypical cerebral dominance for speech-recognition-in-noise.

## Conclusion

This research project primarily aimed to develop a sensitive, reliable and time-efficient clinical tool—the *MAPID-A*—to measure speech-recognition-in-noise performance and SRM in very young children. This key objective has been fulfilled, as shown by the narrow *CI*s of the mean aSNR-50% and the high within-participant test-retest reliability among the young (age: 4–5 years) participants. This validated sensitive and reliable tool appears very likely to be useful as a sensitive speech-recognition-in-noise behavioral marker reflective of the neurophysiological development of the auditory neural pathways. SRM is a collective measure of the benefits of head shadow and binaural unmasking, which, according to recent research studies, has a much longer developmental time course before it achieves an adult level than was previously speculated. The *MAPID-A* offers a valuable opportunity to track the subtle within-participant development in speech-recognition-in-noise and SRM in young children. The *MAPID-A* offers a new potential to behaviorally explore the “antimasking” effect which has not previously been investigated thoroughly in young children. The *MAPID-A* has the potential to provide a new window to identify young children’s communication disorders which are associated with atypical cerebral dominance for speech-recognition-in-noise such as auditory processing disorders and language-learning disorders, following validation in these clinical populations.
